# Multi-Omics Studies towards Novel Modulators of Influenza A Virus–Host Interaction

**DOI:** 10.3390/v8100269

**Published:** 2016-09-29

**Authors:** Sandra Söderholm, Yu Fu, Lana Gaelings, Sergey Belanov, Laxman Yetukuri, Mikhail Berlinkov, Anton V. Cheltsov, Simon Anders, Tero Aittokallio, Tuula A. Nyman, Sampsa Matikainen, Denis E. Kainov

**Affiliations:** 1 Institute of Biotechnology, University of Helsinki, Helsinki 00014, Finland; sandra.soderholm@helsinki.fi; 2 Finnish Institute of Occupational Health, Helsinki 00250, Finland; sampsa.matikainen@helsinki.fi; 3 Institute for Molecular Medicine Finland (FIMM), University of Helsinki, Helsinki 00014, Finland; yu.fu@helsinki.fi (Y.F.); lgaelings@stud.hs-bremen.de (L.G.); sergei.belanov@helsinki.fi (S.B.); yetulax@mappi.helsinki.fi (L.Y.); simon.anders@fimm.fi (S.A.); tero.aittokallio@fimm.fi (T.A.); 4 Institute of Mathematics and Computer Science, Ural Federal University, Yekaterinburg 620083, Russia; berlm@mail.ru; 5 Q-Mol L.L.C. in Silico Pharmaceuticals, San Diego, CA 92037, USA; anton.cheltsov@gmail.com; 6 Department of Mathematics and Statistics, University of Turku, Turku 20014, Finland; 7 Institute of Clinical Medicine, Oslo 0450, Norway; t.a.nyman@medisin.uio.no; 8 Department of Rheumatology, Helsinki University Hospital, University of Helsinki, Helsinki 00015, Finland

**Keywords:** influenza virus, antiviral agent, proteomics, phosphoproteomics, metabolomics, transcriptomics, genomics, virtual ligand screening

## Abstract

Human influenza A viruses (IAVs) cause global pandemics and epidemics. These viruses evolve rapidly, making current treatment options ineffective. To identify novel modulators of IAV–host interactions, we re-analyzed our recent transcriptomics, metabolomics, proteomics, phosphoproteomics, and genomics/virtual ligand screening data. We identified 713 potential modulators targeting 199 cellular and two viral proteins. Anti-influenza activity for 48 of them has been reported previously, whereas the antiviral efficacy of the 665 remains unknown. Studying anti-influenza efficacy and immuno/neuro-modulating properties of these compounds and their combinations as well as potential viral and host resistance to them may lead to the discovery of novel modulators of IAV–host interactions, which might be more effective than the currently available anti-influenza therapeutics.

## 1. Introduction

Approximately 0.18%–0.21% of the amino acids in human influenza virus proteins changes every year due to the error-prone nature of viral RNA-dependent RNA-polymerase [1]. The other source of variation is reassortment, i.e., when the genetic segments of two parental viruses are reasserted in infected cells, giving rise to offspring with a new segment combination. The accumulation of amino acid changes and reassortment events may enable emerging viruses to evade host immunity acquired from previous IAV infections or vaccinations or to develop resistance against available antiviral agents [2].

Almost all IAV strains have already developed resistance to amantadine and rimantadine, due to certain amino acid changes in viral proton-channel M2 [3]. Oseltamivir-, zanamivir-, laninamivir-, and peramivir-resistant IAV strains also emerged and reduced the efficacy of treatment due to mutations in viral neuraminidase (NA) [4]. The critical question remains: what will be the next generation of influenza antivirals that will be less prone to rapid evolution of IAV?

Dozens of novel antiviral drugs are currently under development [5,6]. For example, DAS181, JNJ872, ribavirin, verdinexor, 202 CH65, C05, SaliPhe, nucleozin, geldanamycin, 17-AAG, LJ001, SA-19, fattiviracin, TBHQ, 4C, gemcitabine, ASN2, bortezamib, carfilzomib, C75, 25HC, SNS-032, and MK2206, as well as many other IAV- and host cell-directed agents, are currently under pre-clinical or clinical investigations [5,6,7,8,9]. Some of these, or other antiviral agents under development, could replace the conventional anti-influenza therapeutics in the near future. However, more information about virus-host interactions is needed in order to improve the treatment options for viral diseases. Nowadays, various omics techniques can successfully be used for retrieving information about virus–host interactions at genetic, transcriptional, translational, post-translational and metabolic levels. The integration of this data could be utilized for the identification and development of modulators of infection and potential antiviral drugs. This review attempts to summarize the results of combining transcriptomics, proteomics, phosphoproteomics, metabolomics, and genomics data for identifying potential cellular targets in IAV–host infection.

## 2. Combination of Various Omics Techniques Identifies Potential Novel Modulators of IAV–Host Interaction

**Figure 1:**
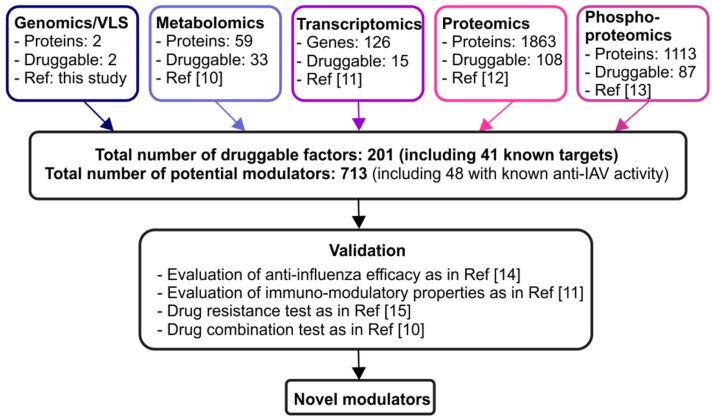
Discovery pipeline for novel modulators of IAV–host interactions. Altogether 713 potential modulators of virus-host interactions derived from the analysis of our recent omics studies [10,11,12,13]. These molecules should be first evaluated in vitro using antiviral efficacy assays, and then in animal models as described in our previous studies [14]. The immuno-modulatory effects of these drugs should also be studied, followed by drug resistant tests as in ref. [15]. In addition, combinations of some of these compounds should be tested, to decrease their toxicity and increase efficacy as described in ref. [10].

Here, we expand the list of potential druggable viral and host targets by re-analyzing our recent transcriptomics, proteomics, phosphoproteomics, metabolomics, and genomics/virtual chemical screening data (Figure 1).

In particular, our recent transcriptomics study identified a total of 126 genes which were up- or down-regulated greater than four-fold in A/Udorn/1972(H3N2) or A/WSN/1933(H1N1) virus infected human peripheral blood mononuclear cells (PBMC)-derived macrophages compared to mock-infected cells at 8 h post infection (*p* < 0.05) [10]. The most significant canonical pathways specifically associated with virus infections, according to gene set enrichment analysis (GSEA; www.broadinstitute.org/gsea, [16]), were interferon-α, -β, and -γ signaling, cytokine signaling, cytokine-cytokine receptor interaction, and cytosolic DNA-sensing pathway. Next, we searched for genes that encode proteins and for which potent chemical/synthetic inhibitors are available, based on the Drug Bank and Drug Gene Interaction Database (http://www.drugbank.ca/; dgidb.genome.wustl.edu/) [17,18]. In this transcriptomic analyses, we identified 15 proteins, which can be targeted with 53 compounds (Table S1).

We also performed quantitative subcellular proteome and secretome studies using human PBMC-derived macrophages and the influenza A/Udorn/1972 strain [12]. We identified 3477 host proteins and reliably quantified 2466 of these proteins using the isobaric tags for relative and absolute quantitation (iTRAQ) technique. In total, 1321 proteins were differentially expressed in the intracellular fractions (fold change ≥ 1.5 or ≤ 0.67) and 544 in the secretome (fold change ≥ 3) as a result of infection. We again searched for druggable proteins among 1865 candidates, using the Drug Bank and Drug Gene Interaction Database [17,18]. We found 108 proteins, which could be targeted with 346 compounds (Table S1). Interestingly, five of these proteins (TNF, CXCL10, CCL3, NAMPT, CCL8) were also found among the druggable targets identified in our transcriptomics study.

We also performed phosphoproteomics profiling of human PBMC-derived macrophages infected with A/Udorn/1972 virus at 6 h post infection [13]. Our analysis identified 1675 phosphoproteins in mock and IAV-infected human macrophages. The phosphorylation of 1113 of these proteins was altered upon infection. We searched for proteins, for which chemical/synthetic inhibitors are available using the Drug Bank and Drug Gene Interaction Database [17,18]. We found 87 phosphoproteins that could be targeted by a total of 382 compounds (Table S1). Among these proteins, there were several cyclin-dependent kinases. Our efficacy studies showed that cyclin-dependent kinase inhibitor SNS-032 efficiently inhibited influenza virus infection in vitro and in vivo [11,13]. Interestingly, 38 druggable proteins identified by phosphoproteomics were also identified in our proteomics study (Table S1).

We have also analyzed the metabolic profiles of PBMC-derived macrophages infected with A/Udorn/1972 or A/WSN/1933 strains for 24 h with LC-MS/MS [10]. In particular, we found that the level of tryptophan was decreased and the level of its oxidation product, l-kynurenine, was elevated. This suggested that tryptophan catabolism was activated during IAV infection. Interestingly, in our transcriptomics study, levels of indoleamine 2,3-dioxygenase (IDO), which catalyzes tryptophan oxidation, was increased 32-fold in IAV-infected macrophages in comparison with the mock macrophages. Similarly, the levels of adenosine, adenine, inosine, inositol monophosphate, and xanthine were altered in IAV-infected macrophages, suggesting that purine metabolism was modulated by IAV infection. In line with the metabolomics results, our transcriptomics experiments showed that the expression of NT5C3, PDE4B, PNPT1, GMPR, ENTPD3, and NUDT2 genes (that are involved in purine metabolism) was up-regulated in response to infection. We also observed alterations in glutathione, nitrogen, arginine and proline, alanine, asparagine and glutamine, histamine, cysteine and methionine metabolic pathways. The molecules (which are all enzymes) identified in the metabolomics study [10] and which are involved in these pathways, were manually examined in the KEGG database [19]. Several compounds targeting these enzymes were then identified using the Drug Bank database [17]. Altogether, we found 33 potential targets for 102 compounds (Table S1).

We have also performed a genomics/virtual chemical screening (VLS) study using available human influenza A(H3N2) and A(H1N1)pdm09 virus sequences, high-resolution IAV protein structures, and a library of FDA-approved drugs. We first downloaded 4983 whole-genome sequences of influenza A(H1N1)pdm09 and 6385 sequences of influenza A(H3N2) strains from Influenza Virus Resource and Global Initiative on Sharing Avian Influenza Data databases (http://www.ncbi.nlm.nih.gov/genomes/FLU/FLU.html; http://platform.gisaid.org/). We converted the nucleotide sequences to protein sequences. The protein sequences were aligned and similarity rates for each amino acid in the alignments were calculated. We used available X-ray and NMR structures of influenza proteins from the protein databank (http://www.rcsb.org/) to mark highly conserved amino acids (see [10] for details). We identified 25 highly conserved sites on influenza proteins. To identify allosteric and cryptic binding sites for potential influenza antivirals, in addition to known active sites and binding pockets, we applied the Q-MOL molecular surface scanning methodology [8]. Q-MOL allows identification of “hot” spots on the molecular surface of a protein (http://q-mol.com/). Briefly, minimized 3D molecular structures of the individual 20 amino acids were used as probes to systematically scan the molecular surface of a protein target. During the scan, non-bonded interactions were evaluated between an amino acid probe and protein residues in proximity of a probe. This methodology allowed the detection of excess energy stored on the surface of a protein. This excess surface energy makes it possible for small molecules and other ligands to specifically bind to protein targets at a particular spot. We identified several hot spots for each target protein, but focused only on two that overlapped with evolutionary conserved sites on the proton channel M2 and polymerase subunit PA (PDB IDs: 2RLF, 4WSB). We performed VLS for these two docking sites using Q-MOL and a library of FDA approved drugs (in total 3655 ligands). After VLS, the ligands were ranked by relative binding energy, sorted, and the seven best hits per target were visually inspected and selected (Table S1).

Altogether, we identified 201 cellular and viral proteins for which 713 inhibitors are available. Interestingly, 41 of the 199 druggable cellular factors were shown to be implicated in IAV infection (Table S2) [10,20,21,22,23,24,25,26,27,28,29,30,31,32,33,34,35,36,37,38,39,40,41,42,43,44,45,46,47,48,49,50,51,52,53,54,55,56,57,58,59,60,61,62,63,64,65,66,67,68,69,70,71,72,73,74,75,76,77,78,79,80,81,82,83,84,85,86,87,88,89,90,91,92]. Moreover, anti-influenza activity was tested for 48 of 713 agents (Table S3) [11,91,93,94,95,96,97,98,99,100,101,102,103,104,105,106,107,108,109,110,111,112,113,114,115,116,117,118,119,120,121,122,123,124,125,126,127,128,129,130,131,132,133,134,135,136,137,138,139,140,141,142,143,144,145,146,147,148,149,150,151,152,153,154,155,156,157,158,159,160,161,162,163,164,165,166,167]. In particular, anti-influenza activity was reported for benzbromarone, ambraxol and tannic acid [168,169,170]. Kinase inhibitors, such as dinaciclib, flavopiridol, SNS-032, and MK2206, and TNF inhibitors, such as etanercept, adalimumab and infliximab, as well as a lipid-lowering simvastatin and antibacterial vancomycin, rifampicin, and erythromycin, were also reported to possess anti-IAV activity [13,104,107,108,116,128,129,147,166,170]. Interestingly, some of the identified inhibitors could be used for treatment of pain and inflammation associated with severe infections [96] (Patent US 20130123345 A1). Our multi-omics studies, however, did not identify some known inhibitors of IAV–host cell interactions, including Mcl1, RNR, Bcl-xL, and Top1 inhibitors [14,171,172]. Moreover, none of the druggable host targets were identified in more than two omics studies. These could be due to the fact that we used macrophages isolated from different donors and the studies were performed at different time points post-IAV infection using different influenza strains.

**Figure 2:**
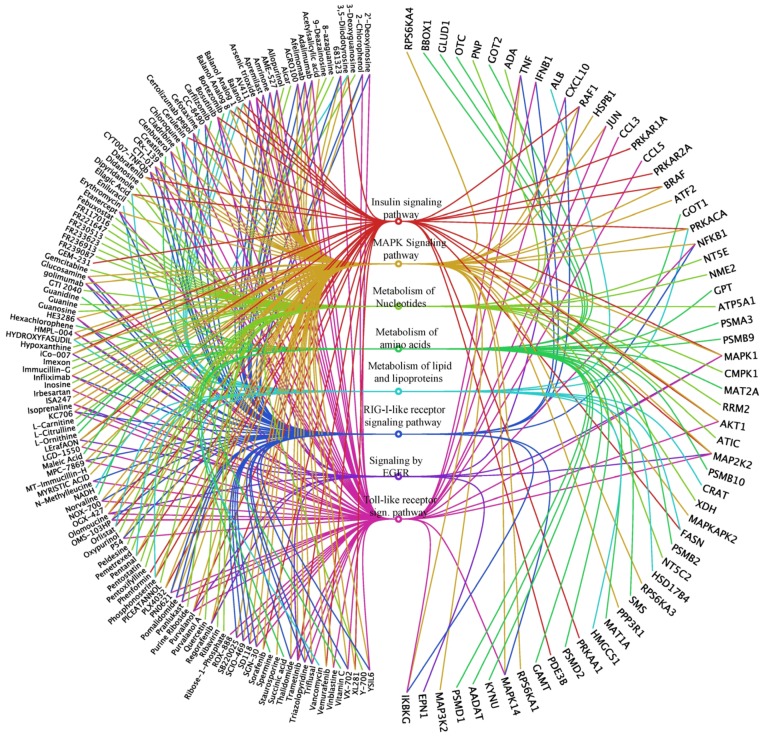
An “eye diagram” showing representative pathways, proteins and compounds, which can be potentially used for modulation of IAV–host interactions. The signaling/metabolic pathways are represented as various colored circles in the middle of the eye diagram. The colored curved lines originating from the pathways connect corresponding compounds on left to target proteins on right. Only the compounds that have shorter names are included for the sake of clarity.

Importantly, based on our omics studies we identified 665 small molecules that target the identified genes/proteins. These compounds might represent novel anti-influenza agents. Based on their target protein annotations, they were clustered into signaling/metabolic pathways by searching KEGG and Reactome databases using DAVID functional annotation tools [173]. The representative pathways, the small molecules and their target proteins are visualized in Figure 2 as an “eye diagram” [174]. Especially interesting are the compounds identified to target proteins/genes identified in at least two omics studies (Table S1). These include a NAMPT inhibitor, GMX1777; a CANX inhibitor, tenecteplase; an ALOX5AP (FLAP) inhibitor, AM103; a NCF2 inhibitor, dextromethorphan; an IGF2R inhibitor, mecasermin; ICAM1 inhibitors, natalizumab and hyaluronic acid; a TPMT inhibitor, olsalazine; and FASN inhibitors, cerulenin and orlistat [175,176,177,178,179]. These small molecules should be first evaluated in vitro using antiviral efficacy assays, and then in animal models as described before. The immuno-modulatory effects of these drugs should also be studied, followed by drug resistant tests [11,15]. In addition, combinations of some of these drugs could be tested, to decrease their toxicity and increase the efficacy of combination treatments [10]. Such follow-up studies would allow identification of safe and effective novel anti-IAV agents. We expect that five to ten novel therapeutics or their combinations could emerge and be used in future clinical studies.

## 3. Conclusions

IAVs have evolved mechanisms to disconcert our innate immunity and secure viral replication [8]. IAVs also deceive our adaptive immunity by constantly modifying their proteins [1]. However, our immune system can still limit virus replication and, in the majority of cases, protect us against the development of severe and lethal infections. But there are a substantial number of cases when IAV infection leads to the hospitalization and even the death of the infected individual (www.who.int). Therefore, more precise understanding of the virus–host interplay might reveal vulnerabilities that can be exploited by direct pharmacological interventions to control and treat IAV infections.

Our recent multi-omics studies provide snapshots of IAV–host cell interaction. They identified a total of 201 cellular and viral factors for which 713 targeting agents are available. Importantly, it is known that many of these agents are safe in humans (i.e., data on adverse compound reactions and adverse effects of other treatments in humans are available), because they were originally developed for the treatment of other diseases. Repurposing these compounds for treating IAV infections or lowering neurological symptoms or modulating immunological reactions could save time and resources in the drug development process. Careful evaluation of these compounds would allow identification of the most potent antiviral agents for further clinical studies. Some of these therapeutics may therefore lead to substantial progress in the treatment of IAV infections, and could perhaps be used to control future influenza epidemics and pandemics.

## Supplementary Materials

The following are available online at www.mdpi.com/1999-4915/8/10/269/s1, Table S1: Genes and proteins implicated in influenza A virus-host interactions and potential modulators of these interactions which were identified through our omics studies, Table S2: Druggable host factors of IAV–host interactions which were identified through our omics studies with cross references to other studies, Table S3: Compounds identified through our omics studies with cross references to other studies.

## References

[1] Belanov S.S., Bychkov D., Benner C., Ripatti S., Ojala T., Kankainen M., Kai Lee H., Wei-Tze Tang J., Kainov D.E. (2015). Genome-wide analysis of evolutionary markers of human influenza A(H1N1)pdm09 and A(H3N2) viruses may guide selection of vaccine strain candidates. Genome Biol. Evol..

[2] Rewar S., Mirdha D., Rewar P. (2015). Treatment and prevention of pandemic H1N1 influenza. Ann. Glob. Health.

[3] Kamps B.S., Hoffmann C. Amantadine. www.influenzareport.com/ir/drugs/amanta.htm.

[4] Spanakis N., Pitiriga V., Gennimata V., Tsakris A. (2014). A review of neuraminidase inhibitor susceptibility in influenza strains. Expert Rev. Anti Infect. Ther..

[5] Zumla A., Rao M., Wallis R.S., Kaufmann S.H., Rustomjee R., Mwaba P., Vilaplana C., Yeboah-Manu D., Chakaya J., Ippolito G. (2016). Host-directed therapies for infectious diseases: Current status, recent progress, and future prospects. Lancet Infect. Dis..

[6] McKimm-Breschkin J.L., Fry A.M. (2016). Meeting report: 4th ISIRV antiviral group conference: Novel antiviral therapies for influenza and other respiratory viruses. Antivir. Res..

[7] Loregian A., Mercorelli B., Nannetti G., Compagnin C., Palu G. (2014). Antiviral strategies against influenza virus: Towards new therapeutic approaches. Cell. Mol. Life Sci..

[8] Muller K.H., Kakkola L., Nagaraj A.S., Cheltsov A.V., Anastasina M., Kainov D.E. (2012). Emerging cellular targets for influenza antiviral agents. Trends Pharmacol. Sci..

[9] Vanderlinden E., Naesens L. (2014). Emerging antiviral strategies to interfere with influenza virus entry. Med. Res. Rev..

[10] Fu Y., Gaelings L., Soderholm S., Belanov S., Nandania J., Nyman T.A., Matikainen S., Anders S., Velagapudi V., Kainov D.E. (2016). JNJ872 inhibits influenza a virus replication without altering cellular antiviral responses. Antivir. Res..

[11] Soderholm S., Anastasina M., Islam M.M., Tynell J., Poranen M.M., Bamford D.H., Stenman J., Julkunen I., Sauliene I., de Brabander J.K. (2016). Immuno-modulating properties of saliphenylhalamide, SNS-032, obatoclax, and gemcitabine. Antivir. Res..

[12] Lietzen N., Ohman T., Rintahaka J., Julkunen I., Aittokallio T., Matikainen S., Nyman T.A. (2011). Quantitative subcellular proteome and secretome profiling of influenza A virus-infected human primary macrophages. PLoS Pathog..

[13] Soderholm S., Kainov D., Ohman T., Denisova O., Schepens B., Kulesskiy E., Imanishi S.Y., Corthals G., Hintsanen P., Aittokallio T. (2016). Phosphoproteomics to characterize host response during influenza A virus infection of human macrophages. Mol. Cell. Proteom..

[14] Denisova O.V., Kakkola L., Feng L., Stenman J., Nagaraj A., Lampe J., Yadav B., Aittokallio T., Kaukinen P., Ahola T. (2012). Obatoclax, saliphenylhalamide, and gemcitabine inhibit influenza A virus infection. J. Biol. Chem..

[15] Denisova O.V., Soderholm S., Virtanen S., von Schantz C., Bychkov D., Vashchinkina E., Desloovere J., Tynell J., Ikonen N., Theisen L.L. (2014). Akt inhibitor MK2206 prevents influenza pH1N1 virus infection in vitro. Antimicrob. Agents Chemother..

[16] Subramanian A., Tamayo P., Mootha V.K., Mukherjee S., Ebert B.L., Gillette M.A., Paulovich A., Pomeroy S.L., Golub T.R., Lander E.S. (2005). Gene set enrichment analysis: A knowledge-based approach for interpreting genome-wide expression profiles. Proc. Natl. Acad. Sci. USA.

[17] Law V., Knox C., Djoumbou Y., Jewison T., Guo A.C., Liu Y., Maciejewski A., Arndt D., Wilson M., Neveu V. (2014). DrugBank 4.0: Shedding new light on drug metabolism. Nucleic Acids Res..

[18] Griffith M., Griffith O.L., Coffman A.C., Weible J.V., McMichael J.F., Spies N.C., Koval J., Das I., Callaway M.B., Eldred J.M. (2013). DGidb: Mining the druggable genome. Nat. Methods.

[19] Kanehisa M., Sato Y., Kawashima M., Furumichi M., Tanabe M. (2016). KEGG as a reference resource for gene and protein annotation. Nucleic Acids Res..

[20] Acharya B., Thapa K. (2016). Indoor staying during winter season makes people more susceptible to flu. J. Nepal Health Res. Counc..

[21] Yu M., Wang Q., Qi W., Zhang K., Liu J., Tao P., Ge S., Liao M., Ning Z. (2016). Expression of inflammation-related genes in the lung of BALB/c mice response to H7N9 influenza A virus with different pathogenicity. Med. Microbiol. Immunol..

[22] Meng J., Liu X., Zhang P., Li D., Xu S., Zhou Q., Guo M., Huai W., Chen X., Wang Q. (2016). Rb selectively inhibits innate IFN-beta production by enhancing deacetylation of IFN-beta promoter through HDAC1 and HDAC8. J. Autoimmun..

[23] Huang L., Ou R., Rabelo de Souza G., Cunha T.M., Lemos H., Mohamed E., Li L., Pacholczyk G., Randall J., Munn D.H. (2016). Virus infections incite pain hypersensitivity by inducing indoleamine 2,3 dioxygenase. PLoS Pathog..

[24] Blank T., Detje C.N., Spiess A., Hagemeyer N., Brendecke S.M., Wolfart J., Staszewski O., Zoller T., Papageorgiou I., Schneider J. (2016). Brain endothelial- and epithelial-specific interferon receptor chain 1 drives virus-induced sickness behavior and cognitive impairment. Immunity.

[25] Marion T., Elbahesh H., Thomas P.G., DeVincenzo J.P., Webby R., Schughart K. (2016). Respiratory mucosal proteome quantification in human influenza infections. PLoS ONE.

[26] Valkenburg S.A., Josephs T.M., Clemens E.B., Grant E.J., Nguyen T.H., Wang G.C., Price D.A., Miller A., Tong S.Y., Thomas P.G. (2016). Molecular basis for universal HLA-A*0201-restricted CD8+ T-cell immunity against influenza viruses. Proc. Natl. Acad. Sci. USA.

[27] Arora S., Lim W., Bist P., Perumalsamy R., Lukman H.M., Li F., Welker L.B., Yan B., Sethi G., Tambyah P.A. (2016). Influenza A virus enhances its propagation through the modulation of Annexin-A1 dependent endosomal trafficking and apoptosis. Cell Death Differ..

[28] Nagesh P.T., Husain M. (2016). Influenza A virus dysregulates host histone deacetylase 1 that inhibits viral infection in lung epithelial cells. J. Virol..

[29] Cao Z., Zhou Y., Zhu S., Feng J., Chen X., Liu S., Peng N., Yang X., Xu G., Zhu Y. (2016). Pyruvate carboxylase activates the RIG-I-like receptor-mediated antiviral immune response by targeting the MAVS signalosome. Sci. Rep..

[30] Pedersen S.R., Christensen J.P., Buus S., Rasmussen M., Korsholm K.S., Nielsen M., Claesson M.H. (2016). Immunogenicity of HLA class I and II double restricted influenza A-derived peptides. PLoS ONE.

[31] Park J.H., Park E.B., Lee J.Y., Min J.Y. (2016). Identification of novel membrane-associated prostaglandin E synthase-1 (mPGES-1) inhibitors with anti-influenza activities in vitro. Biochem. Biophys. Res. Commun..

[32] Sugiyama M.G., Gamage A., Zyla R., Armstrong S.M., Advani S., Advani A., Wang C., Lee W.L. (2016). Influenza virus infection induces platelet-endothelial adhesion which contributes to lung injury. J. Virol..

[33] Zhang S., Tian H., Cui J., Xiao J., Wang M., Hu Y. (2016). The C-Jun N-terminal kinase (JNK) is involved in H5N1 influenza A virus RNA and protein synthesis. Arch. Virol..

[34] Othumpangat S., Noti J.D., McMillen C.M., Beezhold D.H. (2016). ICAM-1 regulates the survival of influenza virus in lung epithelial cells during the early stages of infection. Virology.

[35] Aeffner F., Woods P.S., Davis I.C. (2015). Ecto-5′-nucleotidase CD73 modulates the innate immune response to influenza infection but is not required for development of influenza-induced acute lung injury. Am. J. Physiol. Lung Cell. Mol. Physiol..

[36] Checconi P., Salzano S., Bowler L., Mullen L., Mengozzi M., Hanschmann E.M., Lillig C.H., Sgarbanti R., Panella S., Nencioni L. (2015). Redox proteomics of the inflammatory secretome identifies a common set of redoxins and other glutathionylated proteins released in inflammation, influenza virus infection and oxidative stress. PLoS ONE.

[37] Wang J., Li Q., Xie J., Xu Y. (2015). Cigarette smoke inhibits BAFF expression and mucosal immunoglobulin a responses in the lung during influenza virus infection. Respir. Res..

[38] Fox J.M., Crabtree J.M., Sage L.K., Tompkins S.M., Tripp R.A. (2015). Interferon lambda upregulates IDO1 expression in respiratory epithelial cells after influenza virus infection. J. Interferon Cytokine Res..

[39] Chen Y., Zhou J., Cheng Z., Yang S., Chu H., Fan Y., Li C., Wong B.H., Zheng S., Zhu Y. (2015). Functional variants regulating LGALS1 (Galectin 1) expression affect human susceptibility to influenza A(H7N9). Sci. Rep..

[40] Bian J.R., Nie W., Zang Y.S., Fang Z., Xiu Q.Y., Xu X.X. (2014). Clinical aspects and cytokine response in adults with seasonal influenza infection. Int. J. Clin. Exp. Med..

[41] Nita-Lazar M., Banerjee A., Feng C., Amin M.N., Frieman M.B., Chen W.H., Cross A.S., Wang L.X., Vasta G.R. (2015). Desialylation of airway epithelial cells during influenza virus infection enhances pneumococcal adhesion via galectin binding. Mol. Immunol..

[42] Jolly L., Stavrou A., Vanderstoken G., Meliopoulos V.A., Habgood A., Tatler A.L., Porte J., Knox A., Weinreb P., Violette S. (2014). Influenza promotes collagen deposition via alphaVbeta6 integrin-mediated transforming growth factor beta activation. J. Biol. Chem..

[43] Tung C.Y., Lewis D.E., Han L., Jaja M., Yao S., Li F., Robertson M.J., Zhou B., Sun J., Chang H.C. (2014). Activation of dendritic cell function by soypeptide lunasin as a novel vaccine adjuvant. Vaccine.

[44] Oguin T.H., Sharma S., Stuart A.D., Duan S., Scott S.A., Jones C.K., Daniels J.S., Lindsley C.W., Thomas P.G., Brown H.A. (2014). Phospholipase D facilitates efficient entry of influenza virus, allowing escape from innate immune inhibition. J. Biol. Chem..

[45] Hrincius E.R., Liedmann S., Anhlan D., Wolff T., Ludwig S., Ehrhardt C. (2014). Avian influenza viruses inhibit the major cellular signalling integrator c-Abl. Cell Microbiol..

[46] Liu S.L., Wu Q.M., Zhang L.J., Wang Z.G., Sun E.Z., Zhang Z.L., Pang D.W. (2014). Three-dimensional tracking of Rab5- and Rab7-associated infection process of influenza virus. Small.

[47] Fang S., Zhang K., Wang T., Wang X., Lu X., Peng B., Wu W., Zhang R., Chen S., Zhang R. (2014). Primary study on the lesions and specific proteins in BEAS-2B cells induced with the 2009 A (H1N1) influenza virus. Appl. Microbiol. Biotechnol..

[48] To E.E., Broughton B.R., Hendricks K.S., Vlahos R., Selemidis S. (2014). Influenza A virus and TLR7 activation potentiate Nox2 oxidase-dependent Ros production in macrophages. Free Radic. Res..

[49] Chesarino N.M., McMichael T.M., Hach J.C., Yount J.S. (2014). Phosphorylation of the antiviral protein interferon-inducible transmembrane protein 3 (IFITM3) dually regulates its endocytosis and ubiquitination. J. Biol. Chem..

[50] Jin S., Li Y., Pan R., Zou X. (2014). Characterizing and controlling the inflammatory network during influenza A virus infection. Sci. Rep..

[51] Fujioka Y., Tsuda M., Nanbo A., Hattori T., Sasaki J., Sasaki T., Miyazaki T., Ohba Y. (2013). A Ca^2+^-dependent signaling circuit regulates influenza A virus internalization and infection. Nat. Commun..

[52] McGuire P.J., Tarasenko T.N., Wang T., Levy E., Zerfas P.M., Moran T., Lee H.S., Bequette B.J., Diaz G.A. (2014). Acute metabolic decompensation due to influenza in a mouse model of ornithine transcarbamylase deficiency. Dis. Model. Mech..

[53] Seo Y.J., Pritzl C.J., Vijayan M., Bomb K., McClain M.E., Alexander S., Hahm B. (2013). Sphingosine kinase 1 serves as a pro-viral factor by regulating viral RNA synthesis and nuclear export of viral ribonucleoprotein complex upon influenza virus infection. PLoS ONE.

[54] Ichihashi T., Asano A., Usui T., Takeuchi T., Watanabe Y., Yamano Y. (2013). Antiviral and antiproliferative effects of canine interferon-λ1. Vet. Immunol. Immunopathol..

[55] Buggele W.A., Krause K.E., Horvath C.M. (2013). Small RNA profiling of influenza A virus-infected cells identifies miR-449B as a regulator of histone deacetylase 1 and interferon β. PLoS ONE.

[56] Naesens L., Guddat L.W., Keough D.T., van Kuilenburg A.B., Meijer J., Vande Voorde J., Balzarini J. (2013). Role of human hypoxanthine guanine phosphoribosyltransferase in activation of the antiviral agent T-705 (favipiravir). Mol. Pharmacol..

[57] Huang C.H., Chen C.J., Yen C.T., Yu C.P., Huang P.N., Kuo R.L., Lin S.J., Chang C.K., Shih S.R. (2013). Caspase-1 deficient mice are more susceptible to influenza A virus infection with PA variation. J. Infect. Dis..

[58] Huang L., Li L., Klonowski K.D., Tompkins S.M., Tripp R.A., Mellor A.L. (2013). Induction and role of indoleamine 2,3 dioxygenase in mouse models of influenza A virus infection. PLoS ONE.

[59] Selemidis S., Seow H.J., Broughton B.R., Vinh A., Bozinovski S., Sobey C.G., Drummond G.R., Vlahos R. (2013). Nox1 oxidase suppresses influenza A virus-induced lung inflammation and oxidative stress. PLoS ONE.

[60] Skovgaard K., Cirera S., Vasby D., Podolska A., Breum S.O., Durrwald R., Schlegel M., Heegaard P.M. (2013). Expression of innate immune genes, proteins and microRNAs in lung tissue of pigs infected experimentally with influenza virus (H1N2). Innate Immun..

[61] Jiang W., Wang Q., Chen S., Gao S., Song L., Liu P., Huang W. (2013). Influenza a virus NS1 induces G0/G1 cell cycle arrest by inhibiting the expression and activity of RhoA protein. J. Virol..

[62] Bauer R.N., Brighton L.E., Mueller L., Xiang Z., Rager J.E., Fry R.C., Peden D.B., Jaspers I. (2012). Influenza enhances caspase-1 in bronchial epithelial cells from asthmatic volunteers and is associated with pathogenesis. J. Allergy Clin. Immunol..

[63] Leung H.S., Li O.T., Chan R.W., Chan M.C., Nicholls J.M., Poon L.L. (2012). Entry of influenza A virus with a alpha2,6-linked sialic acid binding preference requires host fibronectin. J. Virol..

[64] Ioannidis I., McNally B., Willette M., Peeples M.E., Chaussabel D., Durbin J.E., Ramilo O., Mejias A., Flano E. (2012). Plasticity and virus specificity of the airway epithelial cell immune response during respiratory virus infection. J. Virol..

[65] Ma H., Kien F., Maniere M., Zhang Y., Lagarde N., Tse K.S., Poon L.L., Nal B. (2012). Human annexin A6 interacts with influenza a virus protein M2 and negatively modulates infection. J. Virol..

[66] Jost S., Quillay H., Reardon J., Peterson E., Simmons R.P., Parry B.A., Bryant N.N., Binder W.D., Altfeld M. (2011). Changes in cytokine levels and NK cell activation associated with influenza. PLoS ONE.

[67] Zuniga J., Torres M., Romo J., Torres D., Jimenez L., Ramirez G., Cruz A., Espinosa E., Herrera T., Buendia I. (2011). Inflammatory profiles in severe pneumonia associated with the pandemic influenza A/H1N1 virus isolated in Mexico city. Autoimmunity.

[68] Yang M.L., Chen Y.H., Wang S.W., Huang Y.J., Leu C.H., Yeh N.C., Chu C.Y., Lin C.C., Shieh G.S., Chen Y.L. (2011). Galectin-1 binds to influenza virus and ameliorates influenza virus pathogenesis. J. Virol..

[69] Qin G., Liu Y., Zheng J., Ng I.H., Xiang Z., Lam K.T., Mao H., Li H., Peiris J.S., Lau Y.L., Tu W. (2011). Type 1 responses of human Vgamma9Vdelta2 T cells to influenza A viruses. J. Virol..

[70] Carroll T.D., Matzinger S.R., Fritts L., McChesney M.B., Miller C.J. (2011). Memory B cells and CD8^+^ lymphocytes do not control seasonal influenza A virus replication after homologous re-challenge of rhesus macaques. PLoS ONE.

[71] Haidari M., Zhang W., Ganjehei L., Ali M., Chen Z. (2011). Inhibition of MLC phosphorylation restricts replication of influenza virus—A mechanism of action for anti-influenza agents. PLoS ONE.

[72] Allen I.C., Moore C.B., Schneider M., Lei Y., Davis B.K., Scull M.A., Gris D., Roney K.E., Zimmermann A.G., Bowzard J.B. (2011). NLRX1 protein attenuates inflammatory responses to infection by interfering with the RIG-I-MAVS and TRAF6-NF-κB signaling pathways. Immunity.

[73] Nakamura R., Maeda N., Shibata K., Yamada H., Kase T., Yoshikai Y. (2010). Interleukin-15 is critical in the pathogenesis of influenza A virus-induced acute lung injury. J. Virol..

[74] Rowe T., Leon A.J., Crevar C.J., Carter D.M., Xu L., Ran L., Fang Y., Cameron C.M., Cameron M.J., Banner D. (2010). Modeling host responses in ferrets during A/California/07/2009 influenza infection. Virology.

[75] Wang D., Harmon A., Jin J., Francis D.H., Christopher-Hennings J., Nelson E., Montelaro R.C., Li F. (2010). The lack of an inherent membrane targeting signal is responsible for the failure of the matrix (M1) protein of influenza A virus to bud into virus-like particles. J. Virol..

[76] Wahl A., Schafer F., Bardet W., Hildebrand W.H. (2010). HLA class I molecules reflect an altered host proteome after influenza virus infection. Hum. Immunol..

[77] Danishuddin M., Khan S.N., Khan A.U. (2010). Molecular interactions between mitochondrial membrane proteins and the C-terminal domain of PB1-F2: An in silico approach. J. Mol. Model..

[78] Thomas P.G., Dash P., Aldridge J.R., Ellebedy A.H., Reynolds C., Funk A.J., Martin W.J., Lamkanfi M., Webby R.J., Boyd K.L. (2009). The intracellular sensor NLRP3 mediates key innate and healing responses to influenza A virus via the regulation of caspase-1. Immunity.

[79] Melchjorsen J., Kristiansen H., Christiansen R., Rintahaka J., Matikainen S., Paludan S.R., Hartmann R. (2009). Differential regulation of the OASL and OAS1 genes in response to viral infections. J. Interferon Cytokine Res..

[80] Kakugawa S., Shimojima M., Goto H., Horimoto T., Oshimori N., Neumann G., Yamamoto T., Kawaoka Y. (2009). Mitogen-activated protein kinase-activated kinase Rsk2 plays a role in innate immune responses to influenza virus infection. J. Virol..

[81] Chen C., Zhuang X. (2008). Epsin 1 is a cargo-specific adaptor for the clathrin-mediated endocytosis of the influenza virus. Proc. Natl. Acad. Sci. USA.

[82] LeBouder F., Morello E., Rimmelzwaan G.F., Bosse F., Pechoux C., Delmas B., Riteau B. (2008). Annexin II incorporated into influenza virus particles supports virus replication by converting plasminogen into plasmin. J. Virol..

[83] Liu N., Song W., Wang P., Lee K., Chan W., Chen H., Cai Z. (2008). Proteomics analysis of differential expression of cellular proteins in response to avian H9N2 virus infection in human cells. Proteomics.

[84] Link C., Ebensen T., Standner L., Dejosez M., Reinhard E., Rharbaoui F., Guzman C.A. (2006). An SopB-mediated immune escape mechanism of *Salmonella enterica* can be subverted to optimize the performance of live attenuated vaccine carrier strains. Microbes Infect..

[85] Zamarin D., Garcia-Sastre A., Xiao X., Wang R., Palese P. (2005). Influenza virus PB1-F2 protein induces cell death through mitochondrial ANT3 and VDAC1. PLoS Pathog..

[86] Olschlager V., Pleschka S., Fischer T., Rziha H.J., Wurzer W., Stitz L., Rapp U.R., Ludwig S., Planz O. (2004). Lung-specific expression of active Raf kinase results in increased mortality of influenza A virus-infected mice. Oncogene.

[87] Sieczkarski S.B., Whittaker G.R. (2003). Differential requirements of Rab5 and Rab7 for endocytosis of influenza and other enveloped viruses. Traffic.

[88] Chen W., Norbury C.C., Cho Y., Yewdell J.W., Bennink J.R. (2001). Immunoproteasomes shape immunodominance hierarchies of antiviral CD8^+^ T cells at the levels of t cell repertoire and presentation of viral antigens. J. Exp. Med..

[89] Julkunen I., Sareneva T., Pirhonen J., Ronni T., Melen K., Matikainen S. (2001). Molecular pathogenesis of influenza A virus infection and virus-induced regulation of cytokine gene expression. Cytokine Growth Factor Rev..

[90] Swiergiel A.H., Smagin G.N., Johnson L.J., Dunn A.J. (1997). The role of cytokines in the behavioral responses to endotoxin and influenza virus infection in mice: Effects of acute and chronic administration of the interleukin-1-receptor antagonist (IL-1ra). Brain Res..

[91] Akaike T., Ando M., Oda T., Doi T., Ijiri S., Araki S., Maeda H. (1990). Dependence on O_2_^−^ generation by xanthine oxidase of pathogenesis of influenza virus infection in mice. J. Clin. Invest..

[92] Pierson D., Knight V., Hansard P., Chan E. (1976). Hepatic carbamyl phosphate synthetase and ornithine transcarbamylase in mouse influenze A and influenze B infection. Proc. Soc. Exp. Biol. Med..

[93] Basaran E., Karakucuk-Iyidogan A., Schols D., Oruc-Emre E.E. (2016). Synthesis of novel chiral sulfonamide-bearing 1,2,4-triazole-3-thione analogs derived from d- and l-phenylalanine esters as potential anti-influenza agents. Chirality.

[94] Akpovwa H. (2016). Chloroquine could be used for the treatment of filoviral infections and other viral infections that emerge or emerged from viruses requiring an acidic pH for infectivity. Cell Biochem. Funct..

[95] Hyuga S., Hyuga M., Oshima N., Maruyama T., Kamakura H., Yamashita T., Yoshimura M., Amakura Y., Hakamatsuka T., Odaguchi H. (2016). Ephedrine alkaloids-free ephedra herb extract: A safer alternative to ephedra with comparable analgesic, anticancer, and anti-influenza activities. J. Nat. Med..

[96] Wu W., Li R., Li X., He J., Jiang S., Liu S., Yang J. (2016). Quercetin as an antiviral agent inhibits influenza A virus (IAV) entry. Viruses.

[97] Chen X.X., Zhou H.X., Qi W.B., Ning Z.Y., Ma Y.J., Li Y.L., Wang G.C., Chen J.X. (2015). Antiviral effects of the combination of glycyrrhizin and ribavirin against influenza a H1N1 virus infection in vivo. Yao Xue Xue Bao.

[98] Lu Y., Hardes K., Dahms S.O., Bottcher-Friebertshauser E., Steinmetzer T., Than M.E., Klenk H.D., Garten W. (2015). Peptidomimetic furin inhibitor MI-701 in combination with oseltamivir and ribavirin efficiently blocks propagation of highly pathogenic avian influenza viruses and delays high level oseltamivir resistance in MDCK cells. Antivir. Res..

[99] Wu L., Dai J., Zhao X., Chen Y., Wang G., Li K. (2015). Chloroquine enhances replication of influenza a virus A/WSN/33 (H1N1) in dose-, time-, and MOI-dependent manners in human lung epithelial cells A549. J. Med. Virol..

[100] Cai Y., Li Y.F., Tang L.P., Tsoi B., Chen M., Chen H., Chen X.M., Tan R.R., Kurihara H., He R.R. (2015). A new mechanism of vitamin C effects on A/FM/1/47(H1N1) virus-induced pneumonia in restraint-stressed mice. BioMed Res. Int..

[101] Ammer E., Nietzsche S., Rien C., Kuhnl A., Mader T., Heller R., Sauerbrei A., Henke A. (2015). The anti-obesity drug orlistat reveals anti-viral activity. Med. Microbiol. Immunol..

[102] Yeganeh B., Ghavami S., Kroeker A.L., Mahood T.H., Stelmack G.L., Klonisch T., Coombs K.M., Halayko A.J. (2015). Suppression of influenza A virus replication in human lung epithelial cells by noncytotoxic concentrations bafilomycin A1. Am. J. Physiol. Lung Cell. Mol. Physiol..

[103] Zhu H., Shi X., Ju D., Huang H., Wei W., Dong X. (2014). Anti-inflammatory effect of thalidomide on H1N1 influenza virus-induced pulmonary injury in mice. Inflammation.

[104] Mehrbod P., Hair-Bejo M., Tengku Ibrahim T.A., Omar A.R., El Zowalaty M., Ajdari Z., Ideris A. (2014). Simvastatin modulates cellular components in influenza A virus-infected cells. Int. J. Mol. Med..

[105] Coulombe F., Jaworska J., Verway M., Tzelepis F., Massoud A., Gillard J., Wong G., Kobinger G., Xing Z., Couture C. (2014). Targeted prostaglandin E_2_ inhibition enhances antiviral immunity through induction of type I interferon and apoptosis in macrophages. Immunity.

[106] Shahiduzzaman M., Ezatti P., Xin G., Coombs K.M. (2014). Proteasomal serine hydrolases are up-regulated by and required for influenza virus infection. J. Proteome Res..

[107] Shi X., Zhou W., Huang H., Zhu H., Zhou P., Zhu H., Ju D. (2013). Inhibition of the inflammatory cytokine tumor necrosis factor-α with etanercept provides protection against lethal H1N1 influenza infection in mice. Crit. Care.

[108] Sheng Y., Zhang C., Qiu X., Zheng W., Ruan J., Shao Y. (2013). Exploring the molecular mechanism of action between drug and RNA polymerase based on partially-resolved spatial structures. Curr. Comput. Aided Drug Des..

[109] Alsuwaidi A.R., Almarzooqi S., Albawardi A., Benedict S., Kochiyil J., Mustafa F., Hartwig S.M., Varga S.M., Souid A.K. (2013). Cellular bioenergetics, caspase activity and glutathione in murine lungs infected with influenza A virus. Virology.

[110] Park S., Kim J.I., Lee I., Lee S., Hwang M.W., Bae J.Y., Heo J., Kim D., Han S.Z., Park M.S. (2013). Aronia melanocarpa and its components demonstrate antiviral activity against influenza viruses. Biochem. Biophys. Res. Commun..

[111] Gluck B., Schmidtke M., Walther M., Meerbach A., Wutzler P. (2013). Simvastatin treatment showed no prophylactic effect in influenza virus-infected mice. J. Med. Virol..

[112] Kim Y., Kim H., Bae S., Choi J., Lim S.Y., Lee N., Kong J.M., Hwang Y.I., Kang J.S., Lee W.J. (2013). Vitamin C is an essential factor on the anti-viral immune responses through the production of interferon-α/β at the initial stage of influenza A virus (H3N2) infection. Immune Netw..

[113] Kim W., Kim S.H., Huh S.Y., Kong S.Y., Choi Y.J., Cheong H.J., Kim H.J. (2013). Reduced antibody formation after influenza vaccination in patients with neuromyelitis optica spectrum disorder treated with rituximab. Eur. J. Neurol..

[114] Belser J.A., Szretter K.J., Katz J.M., Tumpey T.M. (2013). Simvastatin and oseltamivir combination therapy does not improve the effectiveness of oseltamivir alone following highly pathogenic avian H5N1 influenza virus infection in mice. Virology.

[115] Checconi P., Sgarbanti R., Celestino I., Limongi D., Amatore D., Iuvara A., Alimonti A., Garaci E., Palamara A.T., Nencioni L. (2013). The environmental pollutant cadmium promotes influenza virus replication in MDCK cells by altering their redox state. Int. J. Mol. Sci..

[116] Kumaki Y., Morrey J.D., Barnard D.L. (2012). Effect of statin treatments on highly pathogenic avian influenza H5N1, seasonal and H1N1pdm09 virus infections in BALB/c mice. Future Virol..

[117] Wang S., Zhang J., Ye X. (2012). Protein kinase inhibitor flavopiridol inhibits the replication of influenza virus in vitro. Wei Sheng Wu Xue Bao.

[118] Chen Y.H., Wu K.L., Chen C.H. (2012). Methamphetamine reduces human influenza A virus replication. PLoS ONE.

[119] Ali A., Ibrahim M., Eladl A.E., Saif Y.M., Lee C.W. (2013). Enhanced replication of swine influenza viruses in dexamethasone-treated juvenile and layer turkeys. Vet. Microbiol..

[120] Eisenberg R.A., Jawad A.F., Boyer J., Maurer K., McDonald K., Prak E.T., Sullivan K.E. (2013). Rituximab-treated patients have a poor response to influenza vaccination. J. Clin. Immunol..

[121] Yatmaz S., Seow H.J., Gualano R.C., Wong Z.X., Stambas J., Selemidis S., Crack P.J., Bozinovski S., Anderson G.P., Vlahos R. (2013). Glutathione peroxidase-1 reduces influenza A virus-induced lung inflammation. Am. J. Respir. Cell Mol. Biol..

[122] Hu Y., Jin Y., Han D., Zhang G., Cao S., Xie J., Xue J., Li Y., Meng D., Fan X. (2012). Mast cell-induced lung injury in mice infected with H5N1 influenza virus. J. Virol..

[123] Upadhyay A., Chompoo J., Taira N., Fukuta M., Gima S., Tawata S. (2011). Solid-phase synthesis of mimosine tetrapeptides and their inhibitory activities on neuraminidase and tyrosinase. J. Agric. Food Chem..

[124] Garigliany M.M., Desmecht D.J. (2011). *N*-acetylcysteine lacks universal inhibitory activity against influenza A viruses. J. Negat. Results Biomed..

[125] Uchide N., Toyoda H. (2011). Antioxidant therapy as a potential approach to severe influenza-associated complications. Molecules.

[126] Canestri A., Krivine A., Assoumou L., le Corre M., Rozenberg F., Marcelin A.G., Schneider L., Samri A., Carcelain G., Autran B. (2010). Maraviroc does not affect humoral response to the pandemic influenza A-H1N1V 2009 adjuvanted vaccine in HIV-1-infected patients. AIDS.

[127] Dudek S.E., Luig C., Pauli E.K., Schubert U., Ludwig S. (2010). The clinically approved proteasome inhibitor PS-341 efficiently blocks influenza A virus and vesicular stomatitis virus propagation by establishing an antiviral state. J. Virol..

[128] Ferkolj I. (2009). How to improve the safety of biologic therapy in Crohn’s disease. J. Physiol. Pharmacol..

[129] Serrato V.A., Azevedo V.F., Sabatoski V., Goncalves B.P., Machado D.M. (2013). Influenza H1N1 infection in a patient with psoriatic arthritis in treatment with Adalimumab: A case report. Clin. Rheumatol..

[130] Yamaya M., Shinya K., Hatachi Y., Kubo H., Asada M., Yasuda H., Nishimura H., Nagatomi R. (2010). Clarithromycin inhibits type a seasonal influenza virus infection in human airway epithelial cells. J. Pharmacol. Exp. Ther..

[131] Chase G., Deng T., Fodor E., Leung B.W., Mayer D., Schwemmle M., Brownlee G. (2008). Hsp90 inhibitors reduce influenza virus replication in cell culture. Virology.

[132] Vigerust D.J., McCullers J.A. (2007). Chloroquine is effective against influenza A virus in vitro but not in vivo. Influenza Other Respir. Viruses.

[133] Garozzo A., Tempera G., Ungheri D., Timpanaro R., Castro A. (2007). *N*-acetylcysteine synergizes with oseltamivir in protecting mice from lethal influenza infection. Int. J. Immunopathol. Pharmacol..

[134] Kido H., Okumura Y., Yamada H., Le T.Q., Yano M. (2007). Proteases essential for human influenza virus entry into cells and their inhibitors as potential therapeutic agents. Curr. Pharm. Des..

[135] Li W., Maeda N., Beck M.A. (2006). Vitamin C deficiency increases the lung pathology of influenza virus-infected *gulo*^−/−^ mice. J. Nutr..

[136] Ooi E.E., Chew J.S., Loh J.P., Chua R.C. (2006). In vitro inhibition of human influenza a virus replication by chloroquine. Virol. J..

[137] Kumar P., Khanna M., Srivastava V., Tyagi Y.K., Raj H.G., Ravi K. (2005). Effect of quercetin supplementation on lung antioxidants after experimental influenza virus infection. Exp. Lung Res..

[138] Yokozawa T., Sekiya M., Cho E.J., Kurokawa M., Shiraki K. (2004). Effect of wen-pi-tang extract on lung damage by influenza virus infection. Phytomedicine.

[139] Kumar P., Sharma S., Khanna M., Raj H.G. (2003). Effect of quercetin on lipid peroxidation and changes in lung morphology in experimental influenza virus infection. Int. J. Exp. Pathol..

[140] Ungheri D., Pisani C., Sanson G., Bertani A., Schioppacassi G., Delgado R., Sironi M., Ghezzi P. (2000). Protective effect of *N*-acetylcysteine in a model of influenza infection in mice. Int. J. Immunopathol. Pharmacol..

[141] Sieczkarski S.B., Whittaker G.R. (2002). Influenza virus can enter and infect cells in the absence of clathrin-mediated endocytosis. J. Virol..

[142] Yang B., Yao D.F., Ohuchi M., Ide M., Yano M., Okumura Y., Kido H. (2002). Ambroxol suppresses influenza-virus proliferation in the mouse airway by increasing antiviral factor levels. Eur. Respir. J..

[143] Kim H.K., Jeon W.K., Ko B.S. (2001). Flavanone glycosides from *Citrus junos* and their anti-influenza virus activity. Planta Med..

[144] Kastner S.B., Haines D.M., Archer J., Townsend H.G. (1999). Investigations on the ability of clenbuterol hydrochloride to reduce clinical signs and inflammation associated with equine influenza A infection. Equine Vet. J..

[145] Keller P., Simons K. (1998). Cholesterol is required for surface transport of influenza virus hemagglutinin. J. Cell Biol..

[146] Dolganova A., Sharonov B.P. (1997). Application of various antioxidants in the treatment of influenza. Braz. J. Med. Biol. Res..

[147] Sato K., Suga M., Akaike T., Fujii S., Muranaka H., Doi T., Maeda H., Ando M. (1998). Therapeutic effect of erythromycin on influenza virus-induced lung injury in mice. Am. J. Respir. Crit. Care Med..

[148] Moser C.A., Speaker T.J., Offit P.A. (1997). Effect of microencapsulation on immunogenicity of a bovine herpes virus glycoprotein and inactivated influenza virus in mice. Vaccine.

[149] Yokochi S., Ishiwata Y., Saito H., Ebinuma H., Tsuchiya M., Ishii H. (1997). Stimulation of antiviral activities of interferon by a liver extract preparation. Arzneimittelforschung.

[150] Ochiai H., Sakai S., Hirabayashi T., Shimizu Y., Terasawa K. (1995). Inhibitory effect of bafilomycin A1, a specific inhibitor of vacuolar-type proton pump, on the growth of influenza A and B viruses in MDCK cells. Antivir. Res..

[151] Guinea R., Carrasco L. (1995). Requirement for vacuolar proton-ATPase activity during entry of influenza virus into cells. J. Virol..

[152] Conti G., Portincasa P., Chezzi C. (1995). Cerulenin inhibits production of mature virion particles in chick embryo fibroblasts infected by influenza A viruses. Res. Virol..

[153] Broni B., Julkunen I., Condra J.H., Davies M.E., Berry M.J., Krug R.M. (1990). Parental influenza virion nucleocapsids are efficiently transported into the nuclei of murine cells expressing the nuclear interferon-induced mx protein. J. Virol..

[154] Hagiwara S. (1989). Mechanisms of human platelet aggregation and release reaction induced by influenza virus. Nihon Ketsueki Gakkai Zasshi.

[155] Gorse G.J., Kopp W.C. (1987). Modulation by immunosuppressive agents of peripheral blood mononuclear cell responses to influenza A virus. J. Lab. Clin. Med..

[156] Kozhukharova M.S., Slepushkin A.N., Radeva Kh T., Lavrukhina L.A., Demidova S.A. (1987). Evaluation of dipyridamole efficacy as an agent for preventing acute respiratory viral diseases. Vopr. Virusol..

[157] Versluis D.J., Beyer W.E., Masurel N., Wenting G.J., Weimar W. (1986). Impairment of the immune response to influenza vaccination in renal transplant recipients by cyclosporine, but not azathioprine. Transplantation.

[158] Kuzmov K., Galabov A.S., Radeva K., Kozhukharova M., Milanov K. (1985). Epidemiological trial of the prophylactic effectiveness of the interferon inducer dipyridamole with respect to influenza and acute respiratory diseases. Zhurnal Mikrobiol. Epidemiol. Immunobiol..

[159] Schiltknecht E., Ada G.L. (1985). In vivo effects of cyclosporine on influenza A virus-infected mice. Cell. Immunol..

[160] Bernasconi P., Massera E. (1985). Evaluation of a new pharmaceutical form of nimesulide for the treatment of influenza. Drugs Exp. Clin. Res..

[161] Nugent K.M., Shanley J.D. (1984). Verapamil inhibits influenza A virus replication. Arch. Virol..

[162] Semkow R., Loza-Tulimowska M., Wilczynski J., Krus S. (1984). The dynamics of serum antibodies and metaplasia of the lung respiratory epithelium in influenza-virus-preimmunized mice subjected to immunosuppression. Acta Microbiol. Pol..

[163] Krizanova O., Ciampor F., Veber P. (1982). Influence of chlorpromazine on the replication of influenza virus in chick embryo cells. Acta Virol..

[164] Tonew E., Indulen M.K., Dzeguze D.R. (1982). Antiviral action of dipyridamole and its derivatives against influenza virus A. Acta Virol..

[165] Pan Y.T., Elbein A.D. (1982). The formation of lipid-linked oligosaccharides in Madin-Darby canine kidney cells. Changes in oligosaccharide profiles induced by glucosamine. J. Biol. Chem..

[166] Hamzehei M., Ledinko N. (1980). Inhibition of influenza a virus replication by rifampicin and selenocystamine. J. Med. Virol..

[167] Theisen L.L., Erdelmeier C.A., Spoden G.A., Boukhallouk F., Sausy A., Florin L., Muller C.P. (2014). Tannins from hamamelis virginiana bark extract: Characterization and improvement of the antiviral efficacy against influenza a virus and human papillomavirus. PLoS ONE.

[168] Fukuoka M., Minakuchi M., Kawaguchi A., Nagata K., Kamatari Y.O., Kuwata K. (2012). Structure-based discovery of anti-influenza virus a compounds among medicines. Biochim. Biophys. Acta.

[169] Nobata K., Fujimura M., Ishiura Y., Myou S., Nakao S. (2006). Ambroxol for the prevention of acute upper respiratory disease. Clin. Exp. Med..

[170] Perwitasari O., Yan X., O’Donnell J., Johnson S., Tripp R.A. (2015). Repurposing kinase inhibitors as antiviral agents to control influenza A virus replication. Assay Drug Dev. Technol..

[171] Kakkola L., Denisova O.V., Tynell J., Viiliainen J., Ysenbaert T., Matos R.C., Nagaraj A., Ohman T., Kuivanen S., Paavilainen H. (2013). Anticancer compound ABT-263 accelerates apoptosis in virus-infected cells and imbalances cytokine production and lowers survival rates of infected mice. Cell Death Dis..

[172] Rialdi A., Campisi L., Zhao N., Lagda A.C., Pietzsch C., Ho J.S., Martinez-Gil L., Fenouil R., Chen X., Edwards M. (2016). Topoisomerase 1 inhibition suppresses inflammatory genes and protects from death by inflammation. Science.

[173] Huang D.W., Sherman B.T., Lempicki R.A. (2009). Bioinformatics enrichment tools: Paths toward the comprehensive functional analysis of large gene lists. Nucleic Acids Res..

[174] Caldas J., Gehlenborg N., Faisal A., Brazma A., Kaski S. (2009). Probabilistic retrieval and visualization of biologically relevant microarray experiments. Bioinformatics.

[175] Stanciu C.N., Penders T.M., Rouse E.M. (2016). Recreational use of dextromethorphan, “Robotripping”—A brief review. Am. J. Addict..

[176] Huang X., MacIsaac R., Thompson J.L., Levin B., Buchsbaum R., Haley E.C., Levi C., Campbell B., Bladin C., Parsons M. (2016). Tenecteplase versus alteplase in stroke thrombolysis: An individual patient data meta-analysis of randomized controlled trials. Int. J. Stroke.

[177] Vitaliti G., Matin N., Tabatabaie O., di Traglia M., Pavone P., Lubrano R., Falsaperla R. (2015). Natalizumab in multiple sclerosis: Discontinuation, progressive multifocal leukoencephalopathy and possible use in children. Expert Rev. Neurother..

[178] Bain G., King C.D., Rewolinski M., Schaab K., Santini A.M., Shapiro D., Moran M., van de Wetering de Rooij S., Roffel A.F., Schuilenga-Hut P. (2010). Pharmacodynamics and pharmacokinetics of AM103, a novel inhibitor of 5-lipoxygenase-activating protein (*FLAP*). Clin. Pharmacol. Ther..

[179] Beauparlant P., Bedard D., Bernier C., Chan H., Gilbert K., Goulet D., Gratton M.O., Lavoie M., Roulston A., Turcotte E. (2009). Preclinical development of the nicotinamide phosphoribosyl transferase inhibitor prodrug GMX1777. Anticancer Drugs.

